# Do invasive quagga mussels alter CO_2_ dynamics in the Laurentian Great Lakes?

**DOI:** 10.1038/srep39078

**Published:** 2016-12-20

**Authors:** Peng Lin, Laodong Guo

**Affiliations:** 1 School of Freshwater Sciences, University of Wisconsin-Milwaukee, 600 East Greenfield Avenue, Milwaukee, WI 53204, USA

## Abstract

The Laurentian Great Lakes have experienced unprecedented ecological and environmental changes, especially after the introduction of invasive quagga mussel (*Dreissena rostriformis bugensis*). While impacts on ecological functions have been widely recognized, the response of carbon dynamics to invasive species remains largely unknown. We report new CO_2_ data showing significant increases in *p*CO_2_ (up to 800 μatm in Lake Michigan) and CO_2_ emission fluxes in most of the Great Lakes compared to those prior to or during the early stage of the colonization of invasive quagga mussels. The increased CO_2_ supersaturation is most prominent in Lakes Huron and Michigan, followed by Lakes Ontario and Erie, but no evident change was observed in Lake Superior. This trend mirrors the infestation extent of invasive quagga mussels in the Great Lakes and is consistent with the decline in primary production and increase in water clarity observed pre- and post-*Dreissena* introduction, revealing a close linkage between invasive species and carbon dynamics. The Great Lakes have become a significant CO_2_ source to the atmosphere, emitting >7.7 ± 1.0 Tg-C annually, which is higher than the organic carbon burial rate in global inland-seas and attesting to the significant role of the Laurentian Great Lakes in regional/global CO_2_ budget and cycling.

Levels of carbon dioxide (CO_2_) not only can serve as an indicator of autotrophic or heterotrophic nature of an aquatic environment^1^, but also can be an important parameter to elucidate calcification and potential pH changes in a water body^2^,^3^,^4^. Due to increasing human consumption of fossil fuels and other anthropogenic activities, atmospheric CO_2_ level has increased from 280 μatm in the 1800 s to levels above 400 μatm in 2015 5. As the most important greenhouse gas on earth, inventory and fluxes of CO_2_ across different reservoirs and its role in the global carbon cycle and thus climate and environmental changes have been the focus of many recent research programs^6^,^7^,^8^.

Unlike the open ocean which contains abundant dissolved inorganic carbon (DIC, usually >2000 μmol/kg) and typically serves as a sink of CO_2_^7^,^9^,^10^,^11^, lakes and inland waters could serve as a source of CO_2_ to the atmosphere^12^,^13^,^14^ and may play an essential role to the local, regional and even global carbon cycles and climate. Recently, carbon cycling and magnitude of CO_2_ fluxes across the air-water interface in lakes and inland waters have received increasing attention^15^,^16^,^17^,^18^, although studies on carbon dynamics in the Great Lakes remain scarce^17^,^19^.

The Laurentian Great Lakes are the largest freshwater system on Earth and receive vast amounts of organic and inorganic carbon from surrounding terrestrial ecosystems. Over the past decades, the Great Lakes have experienced significant ecological and environmental changes due to the introduction of invasive species, notoriously nonindigenous quagga mussels (*Dreissena rostiformis bugensis*), leading to a decrease in primary production and increasing water clarity in the Great Lakes^20^,^21^,^22^,^23^,^24^,^25^. In addition to impacts on foodweb structure and ecological functions, changes in biogeochemical cycles of nutrients have also been documented^26^,^27^,^28^. However, specific changes in carbon dynamics, as well as the impacts and biogeochemical consequences after the colonization of invasive quagga mussels in the Laurentian Great Lakes remain poorly understood. The direction and magnitude of air-lake CO_2_ fluxes after *dreissena* introduction in the Great Lakes are largely unquantified, especially in Lakes Michigan, Huron and Ontario^17^,^29^,^30^,^31^.

To examine the response of carbon dynamics to the introduction of invasive quagga mussels and linkages between invasive species and changes in biogeochemical cycling, open lake water samples were collected from all of the Laurentian Great Lakes, including Lake Superior, Lake Michigan, Lake Huron, Lake Erie and Lake Ontario, during August 2013 (Fig. 1). Furthermore, seasonal water samples were also collected from Lake Michigan between 2013 and 2015. In addition to water isotopic composition (δ^2^H and δ^18^O), total alkalinity (TA), dissolved inorganic carbon (DIC), and pH were measured prior to the evaluation of the partial pressure of CO_2_ (*p*CO_2_) and air-water CO_2_ fluxes.

## Results

### Variations in pH, TA and DIC in the Great Lakes

The Laurentian Great Lakes can be characterized as a high-pH and high-carbonate ecosystem although Lake Superior had a relatively lower pH and carbonate abundance compared to the other Great Lakes (Fig. 2 and Table 1). For example, pH values in Great Lakes waters were typically higher than 8 except for two sampling sites in Lake Superior (Table 1). Lakes Michigan, Erie and Ontario had higher pH values, averaging 8.22 ± 0.04, 8.19 ± 0.08 and 8.20 ± 0.04, respectively, followed by Lake Huron (8.07 ± 0.06) and Lake Superior (7.98 ± 0.05).

Concentrations of DIC and TA typically exceeded the threshold of 1000 μmol/kg except for Lake Superior (Table 1). Values of TA varied from 830 to 2200 μmol/kg for all the Great Lakes, with an average of 843 ± 10 μmol/kg in Lake Superior, 2168 ± 22 μmol/kg in Lake Michigan, 1600 ± 59 μmol/kg in Lake Huron, 1872 ± 22 μmol/kg in Lake Erie and 1815 ± 19 μmol/kg in Lake Ontario (Table 1). Similarly, different DIC concentrations were observed among the five Great Lakes, showing the highest value in Lake Michigan (average of 2065 ± 24 μmol/kg) and lowest values in Lake Superior (average of 793 ± 26 μmol/kg). Generally, these DIC and TA values are higher than those in most freshwater systems (e.g., refs 32, 33 and 34) and comparable to those of global ocean basins^35^ (especially data of Lake Michigan, Table 1S). Our data here are also similar to the long-term data from previous studies in the Great Lakes^36^. High DIC and TA in Great Lakes waters are related to the spatial distribution of limestone and carbonate weathering in the Great Lakes basin. It is the high carbonate abundance that allows the thriving and rapid colonization of the mussel community in the Great Lakes^37^, which in turn has “re-engineered” the lake ecosystem during the past decades^22^,^23^,^24^,^25^.

The surface distribution (Fig. 2) showed that TA, pH and DIC concentration all increased consistently from the upper Great Lakes (i.e., Lake Superior) to Lake Huron and then to the lower Great Lakes (i.e., Lakes Erie and Ontario), similar to the increasing δ^2^H and δ^18^O values in surface waters along the Great Lakes. This is consistent with the general water transport pathway from the upper to the lower Great Lakes, suggesting an accumulative effect on water chemistry in the lower Great Lakes from surrounding riverine inputs^38^. Among the five Great Lakes, Lake Michigan had notably high values for each of the measured parameters including pH, TA, DIC, and water isotopes (Fig. 2 and Table 1), which is related to its high abundance of limestone and the fact that it is a semi-closed basin which allows the accumulative effect of evaporation in Lake Michigan.

### Variations in *p*CO_2_ along the Great Lakes

As shown in Table 1 and Fig. 3, our results consistently showed an evident CO_2_ supersaturation (>398 μatm) for all the Great Lakes, with the highest *p*CO_2_ in Lake Michigan (762 ± 88 μatm) and Lake Huron (774 ± 92 μatm), followed by Lakes Erie (725 ± 126 μatm) and Ontario (647 ± 47 μatm). Even in Lake Superior, with the lowest TA and DIC abundance (both <1000 μmol/kg) and lowest *p*CO_2_ values (461 ± 77 μatm), CO_2_ supersaturation was still evident (Fig. 3). This indicated that surface waters of all the Laurentian Great Lakes can be a net CO_2_ source to the atmosphere, consistent with those observed for other global lakes^16^,^18^.

Additionally, our seasonal *p*CO_2_ data from Lake Michigan between 2013 and 2015 also showed a significant CO_2_ supersaturation during all sampling seasons. For example, average *p*CO_2_ was 762 ± 88 μatm in August 2013, 699 ± 17 μatm in May 2014, 769 ± 124 μatm in October 2014, 815 ± 48 μatm in October 2015, and 944 ± 83 μatm in December 2015 (Fig. 4). Furthermore, CO_2_ supersaturation existed throughout the whole water column of open Lake Michigan although *p*CO_2_ values decreased slightly in the middle water column due to biological uptake (see examples of vertical profiles in October 2015, Fig. 4).

## Discussion

The invasive species, especially filter feeders including zebra and quagga mussels, have been shown to strongly affect the foodweb structure and biogeochemical cycles of nutrients after their introduction to the Great Lakes^21^,^22^,^23^,^24^,^25^. Thus, the equilibrium and interaction of CO_2_ between lake and atmosphere may have been altered during the past decades, particularly after quagga mussels became predominant. As shown in Fig. 3, the *p*CO_2_ values in the Great Lakes observed in 2013 are significantly higher than the median values of *p*CO_2_ observed during the same sampling season between 1983–2006^17^,^30^,^39^ (Table 1), especially for Lakes Michigan (*p* < 0.001), Huron (*p* < 0.001), Erie (*p* = 0.004) and Ontario (*p* = 0.035). Compared to the increase in atmospheric CO_2_ levels from 1983 to 2013 (~60 μatm), the increase in *p*CO_2_ in the water column of the Great Lakes was considerably larger. Taking Lakes Michigan and Huron as examples, during 1983–2006, the average ∆*p*CO_2_ value, the difference in *p*CO_2_ between atmosphere (averaging ~362 μtm during that time periods) and lake waters, was about 118 and 22 μatm for Lakes Michigan and Huron^30^,^39^, respectively. In comparison, ∆*p*CO_2_ values in these two lakes during summer 2013 reached a new high of ~400 μatm, averaging 365 ± 89 and 377 ± 92 μatm for Lake Michigan and Lake Huron, respectively (Table 1), showing an increase of 4 times in ∆*p*CO_2_ values in August between 1983–2006 and 2013 (Fig. 3). For Lakes Erie and Ontario, the increase in *p*CO_2_ values between 1983–2006 and 2013 was also significant, although large errors existed in historical CO_2_ data^30^,^39^ for the shallower lakes with high primary production and anthropogenic influences including seasonal hypoxia and algal bloom, especially in Lake Erie^40^,^41^.

Moreover, our seasonal CO_2_ data and its vertical distribution in Lake Michigan (Fig. 4) further demonstrated the increased CO_2_ supersaturation in the water column. For example, *p*CO_2_ in Lake Michigan during October 2015 ranged from ~600 μatm in subsurface waters to ~770 μatm in deeper waters (Fig. 4). Elevated *p*CO_2_ in deeper waters resulted from the decomposition of organic matter and decreased biological uptake also implied a potential higher CO_2_ emission flux from lake waters when the CO_2_-enriched deeper water is upwelled to surface during winter mixing seasons^42^. Furthermore, our seasonal data clearly show a consistent increase in *p*CO_2_ from summer to fall and then to winter, reaching as high as 944 ± 83 μatm during December 2015; an increase by ~30% compared to other seasons (Fig. 4). Thus, the *p*CO_2_ data obtained during summer 2013 (Fig. 3) might represent the lower limit of CO_2_ supersaturation in the Great Lakes, although other factors could have an influence, such as partial ice-cover during winter.

In contrast to Lake Michigan and other lower Great Lakes, the increase in *p*CO_2_ in Lake Superior during summer between 1983–2006^17^,^30^ and 2013 does not seem to be significant (*p* = 0.665, Fig. 3, Table 1). Nevertheless, increased CO_2_ supersaturation in Lake Michigan and other lower Great Lakes coincides with the distribution and density of invasive quagga mussels among all the Great Lakes. For example, high quagga mussel densities (e.g., >19,000-mussels/m^2^) have been reported for Lake Michigan^22^,^25^,^27^,^43^, but a very low density for Lake Superior. Although zebra mussels arrived into the Great Lakes before quagga messuls, they have much weaker filter ability, and changes in foodweb structure and ecological function were more evident only after quagga mussels became predominant^25^,^44^,^45^,^46^. In addition, quagga mussels did not start blanketing offshore regions of the Great Lakes, such as Lake Michigan until 2005^25^,^44^. Therefore, CO_2_ data collected during 1983–2006^17^,^30^,^39^ can be considered representative of the time period prior to or during the early stage of the colonization of invasive quagga mussels in the Great Lakes. Linking the spatiotemporal variations of quagga mussel population with the changes in the extent of CO_2_ supersaturation during summer sampling time, we hypothesized that increasing CO_2_ supersaturation in the Great Lakes during the past decade has been induced by the colonization of invasive quagga mussels and the subsequent biogeochemical response to that colonization.

Possible pathways and mechanisms to support our hypothesis include (1) decreasing primary production in the Great Lakes after the introduction of invasive quagga mussels^20^,^21^,^22^,^23^,^47^,^48^; (2) increase in water clarity^24^ which enables the light penetration into deeper waters and consequently enhances the photo-degradation of natural organic matter in the water column; and (3) metabolic processes of vast quantities of invasive quagga mussels blanketing lake floor^27^,^49^, although other processes, such as changes in sediment-water processes, coastal erosion and water chemistry after the colonization of quagga mussels, are also important. All these changes are inter-correlated and are the result of the colonization of invasive quagga mussels and all these processes would directly or indirectly favor the accumulation and release of CO_2_ in the water column, resulting in increased CO_2_ abundance in the Great Lakes, especially those heavily infested with quagga mussels (e.g., Lake Michigan^25^). The close coupling between invasive species and carbon dynamics elucidated here in the Great Lakes clearly shows how small invasive quagga mussels could have caused basin-scale changes in carbon dynamics and biogeochemical cycling.

The increase in *p*CO_2_ levels after the colonization of invasive quagga mussels in the Great Lakes evidently exceeded the increase in average atmospheric CO_2_ level from 1983 to 2013 (~60 μatm). Thus, enhanced CO_2_ emission fluxes from lake waters to the atmosphere can be expected. Based on these summer *p*CO_2_ data, the daily CO_2_ emission fluxes ranged from 8.37 to 14.48 mmol-C/m^2^/d with an average of 10.38 ± 2.52 mmol-C/m^2^/d for Lake Michigan and ranged from 7.69 to 13.60 mmol-C/m^2^/d, averaging 10.71 ± 2.61 mmol-C/m^2^/d for Lake Huron (Fig. 5 and Table 1). These CO_2_ emission fluxes were 3 to 4 times higher than those in Lakes Michigan and Huron at the same sampling time during 1983–2006, based on their differences in ∆*p*CO_2_ values. Lakes Erie and Ontario had CO_2_ emission fluxes of 9.35 ± 3.59 mmol-C/m^2^/d and 7.13 ± 1.33 mmol-C/m^2^/d respectively, while an emission flux of 1.84 ± 2.17 mmol-C/m^2^/d was estimated for Lake Superior. Overall, the emission fluxes of CO_2_ in all five Great Lakes follow the order of Huron ≥ Michigan > Erie > Ontario > Superior during summer 2013 (Fig. 5 and Table 1), distinct from the order of Superior > Michigan > Huron > Ontario > Erie observed prior to or during the early stage of quagga mussel colonization^30^,^50^ and further attests to the strong impact of invasive species on carbon cycles in the Laurentian Great Lakes.

Although CO_2_ emission fluxes from the Great Lakes were comparable to or lower than those from small lakes (e.g., refs 51, 52 and 53), the large surface areas still made the Great Lakes a strong CO_2_ source to the atmosphere. We estimated the lake-wide integrated CO_2_ fluxes for each Great Lake and those estimated flux values are 1.5 × 10^8^ mol-C/d for Lake Superior, 6.0 × 10^8^ mol-C/d for Lake Michigan, 6.4 × 10^8^ mol-C/d for Lake Huron, 2.4 × 10^8^ mol-C/d for Lake Erie, and 1.4 × 10^8^ mol-C/d for Lake Ontario (Fig. 5). Collectively, up to (7.7 ± 1.0) × 10^12^ g-C/yr or 7.7 ± 1.0 g-C/yr in the form of CO_2_ can be released to the atmosphere from the Laurentian Great Lakes, which is comparable to the annual DIC flux to the ocean from the Mississippi River and many other world rivers^54^,^55^. This annual CO_2_ emission flux from the Laurentian Great Lakes is also higher than the annual organic carbon burial rate in all inland seas^16^,^56^, and makes up 1% of the annual CO_2_ emission from global deforestation^57^. It should be noted that our estimated CO_2_ emission flux could be the lower limit since it was calculated from summer sampling when *p*CO_2_ levels were lower relative to winter seasons^42^ (e.g., Fig. 4). Consequently, the Laurentian Great Lakes certainly serve as a significant CO_2_ source to the atmosphere especially after the colonization of invasive species, and play an essential role in regional and global CO_2_ budgets and cycling. This is a paradox in that the Great Lakes with similar carbonate abundance and pH as those in global oceans, should be expected to absorb CO_2_ from the atmosphere and become acidified in the face of the accelerating rise of atmospheric CO_2_^58^. However, the Great Lakes have responded actively to the changing ecosystem and are making a positive feedback to climate change, with decreased whiting events (or CaCO_3_ saturation/precipitation) during summer^24^, increased CO_2_ supersaturation and emission fluxes after the introduction of invasive quagga mussels and resultant decrease in primary production, enhanced degradation of natural organic matter due to increased water clarity, and direct CO_2_ release from quagga mussel respiration^27^,^49^.

## Materials and Methods

### Sampling

**Figure 1:**
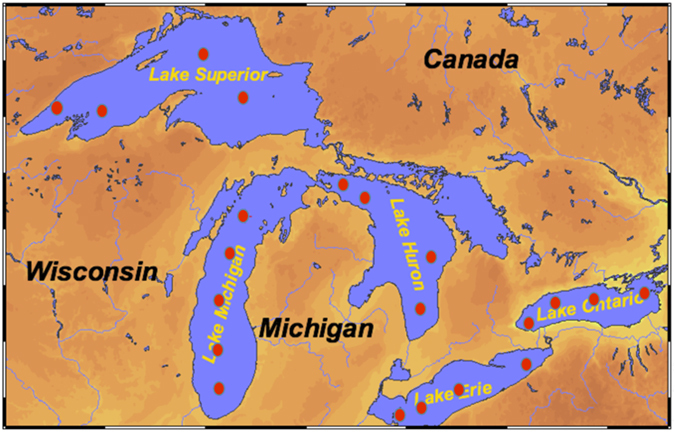
Sampling locations in the Laurentian Great Lakes during August 2013 (The map is created from Ocean Data View (Schlitzer, R., Ocean Data View, odv.awi.de, 2015) and edited on Microsoft PowerPoint 14.5.8 (https://products.office.com/en-us/home).

During August 2013, surface water samples were collected from open lake stations on all of the Great Lakes: including Lake Superior, Lake Michigan, Lake Huron, Lake Erie and Lake Ontario (LS, LM, LH, LE and LO). Specific sample locations are shown in Fig. 1 and Table 1. Additionally, seasonal transect sampling, including a vertical profile at an open lake station (43°11.5362′N; 87°39.9566′W, 104 m depth), was also conducted in open Lake Michigan during 2013 to 2015. Detailed sampling locations can be found in ref. 28. Surface water samples were taken from a hull-mounted, all-Teflon and stainless steel, high-speed pumping system (2 m depth). Hydrographic parameters were recorded with a Hydrolab Datasonde 5A and coordinated with ship’s positioning systems, such as temperature (°C) and chlorophyll-*a* fluorescence (V). Vertical profiles of hydrographic characteristics (SeaBird SBE 25Plus) and Niskin bottle-collected water samples were taken in open Lake Michigan at the offshore station. After collection, samples were saved for pH and total alkalinity (TA) and water isotopes, and filtered through pre-rinsed syringe filters for the measurements of dissolved inorganic carbon (DIC) and dissolved organic carbon (DOC).

### Analysis

Concentrations of DOC were measured on a Shimadzu TOC-L analyzer using the high temperature combustion method^59^. Total dissolved carbon (TDC) was measured on the same TOC analyzer without acidification and sparging. The concentrations of DIC were calculated by the difference between TDC and DOC concentrations^60^. Community consensus seawater reference from the University of Miami for DOC and the certified reference seawater (CRS) from the Scripps Institution of Oceanography for DIC^53^, as well as working standards were measured as samples to ensure data quality. After calibration with three standard solutions (pH = 4.00, 7.00 and 10.00), the pH electrode (Sartorius PB-11) was used to measure the pH. The precision and accuracy of pH was ±0.01. Based on the Gran titration procedure^61^, the sample (~30 mL) for TA was titrated by the CRS-calibrated HCl solution (~0.02 M) to an endpoint pH of 4.5. The results of DIC, DOC and TA are all reported in μmol/kg. Stable hydrogen and oxygen isotopic composition (δ^2^H and δ^18^O) of lake waters was measured on a Picarro cavity ring down spectrometer (L2130-i). Standard mean ocean water (Kona water) was used as a standard for both δ^2^H and δ^18^O with a precision of ±0.02‰.

### Calculations of *p*CO_2_ and flux of CO_2_

*p*CO_2_ was calculated using the CO_2_ program^62^ and measured pH and DIC concentrations or TA under the freshwater option. The CO_2_ fluxes (F) were calculated based on the one-dimension flux model:


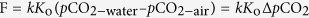


where k (cm/h) is the gas transfer velocity of CO_2_, and the K_o_ (mol/m^3^/atm) is the solubility coefficient of CO_2_ at *in situ* temperature and salinity. Values of *k* and K_o_ were calculated based on the method of ref. 63 and ref. 64, respectively, and the monthly average wind speed in the study area was used for the calculation. The *p*CO_2-water_ and *p*CO_2-air_ are the partial pressures of CO_2_ (in μatm) in surface waters and air, respectively. The global annual averaged surface air *p*CO_2_ for 2013 from NOAA (http://www.esrl.noaa.gov/gmd/) was used as the *p*CO_2-air_ value (398 μatm). When the difference in partial pressure of CO_2_ between water and air (∆*p*CO_2_) is higher than zero, the emission of CO_2_ from surface waters to the atmosphere occurs. In contrast, a negative value of ∆*p*CO_2_ indicates uptake of atmospheric CO_2_ in surface waters.

### Uncertainties of *p*CO_2_ and statistical analysis

**Figure 2:**
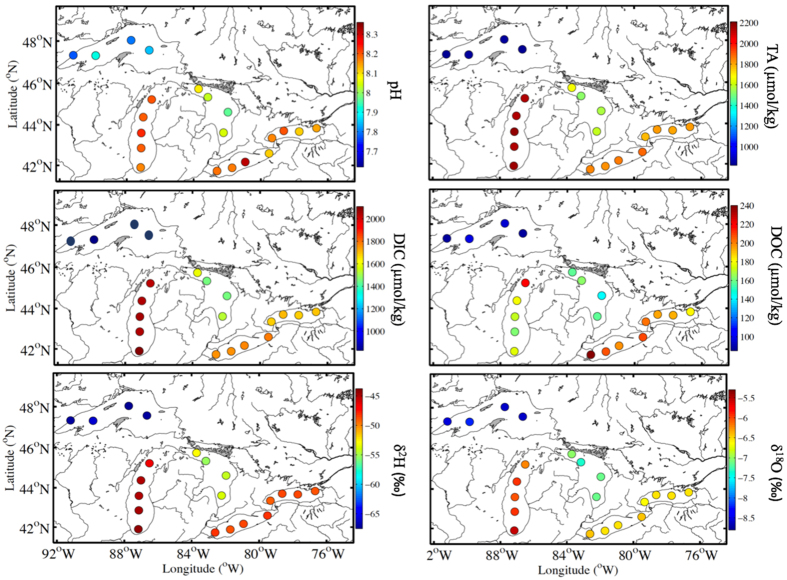
Spatial distributions of surface water pH, total alkalinity (TA), dissolved inorganic carbon (DIC), dissolved organic carbon (DOC) and water isotope composition (δ^2^H and δ^18^O) in the Laurentian Great Lakes during the summer of 2013. The figure is created from MATLAB R2013 (http://www.mathworks.com/products/matlab/index.html?s_tid=gn_loc_drop) and modified by Microsoft PowerPoint 14.5.8 (https://products.office.com/en-us/home).

**Figure 3:**
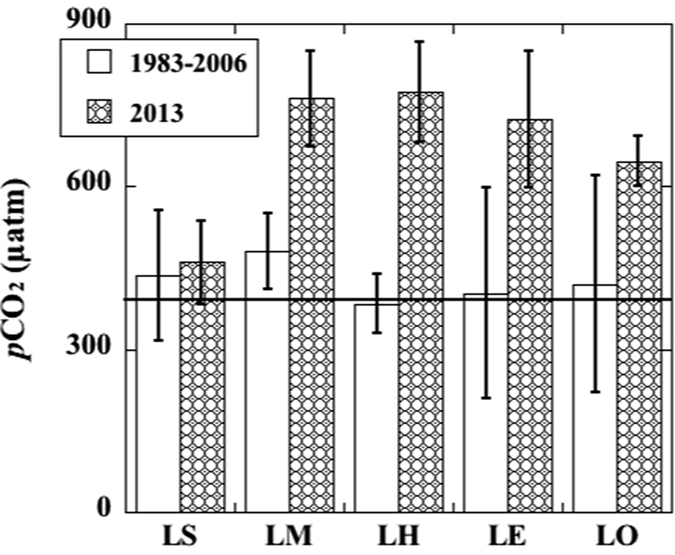
The averaged partial pressure of CO_2_ (*p*CO_2_) in the Laurentian Great Lakes during summer months from 1983–2006 and 2013. LS, LM, LH, LE and LO denote Lakes Superior, Michigan, Huron, Erie and Ontario, respectively. The horizontal line represents the averaged atmospheric *p*CO_2_ value in 2013 (398 μatm, from NOAA at http://www.esrl.noaa.gov/gmd/). Data from 1983–2006 are calculated from surface water pH and alkalinity obtained from the EPA’s biannual Great Lakes surveillance program, which begin from 1983 except for Lake Superior beginning from 1992 (see Urban and Desai (2009)^30^). The *p*CO_2_ values for Lakes Superior, Huron and Erie were derived from the averaged values from previous studies^17^,^30^,^39^, excluding outlier data points.

**Figure 4:**
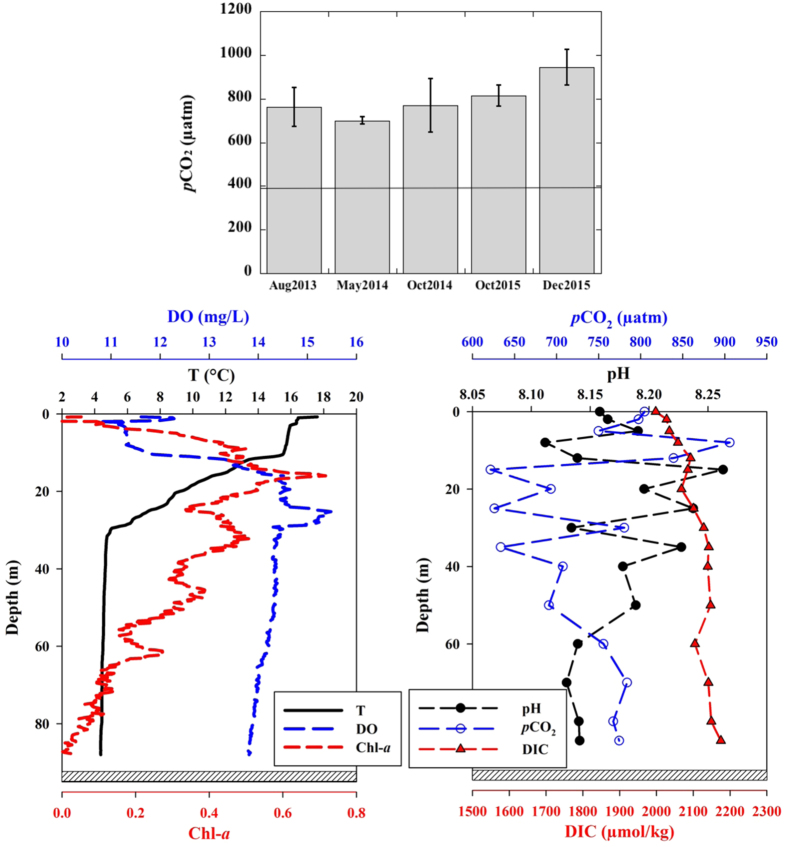
Seasonal variations in partial pressure of CO_2_ (*p*CO_2_) in surface waters of Lake Michigan (upper panel) between 2013 and 2015 and the vertical profiles of *p*CO_2_ and other hydrological data (pH, dissolved inorganic carbon, DIC) in open Lake Michigan during October 2015 (lower panels). The horizontal line represents the averaged atmospheric *p*CO_2_ value (398 μatm) during 2013–2015.

**Figure 5:**
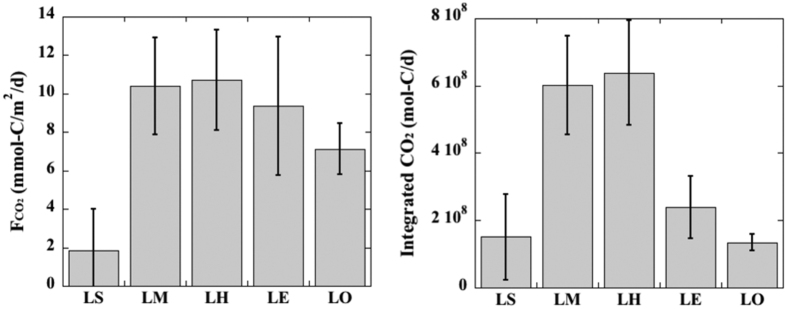
The averaged daily CO_2_ emission fluxes (F_CO2_), and lake-wide integrated CO_2_ emission fluxes in all Laurentian Great Lakes during the summer of 2013, derived from pH-dissolved inorganic carbon (pH-DIC) data pair. LS, LM, LH, LE and LO denote Lakes Superior, Michigan, Huron, Erie and Ontario.

Both DIC-pH and TA-pH pairs were used to derive the *p*CO_2_ values in lake waters. Uncertainties of *p*CO_2_ derived from pH ( ± 0.01) were estimated to be  ± 16 μatm. For the discussion on CO_2_ fluxes, *p*CO_2_ data derived from pH-DIC were used, while data derived from pH-TA were used to evaluate possible contribution from non-carbonate alkalinity^65^. As shown in Table 1, *p*CO_2_ values calculated from pH-TA pair were, on average, 48.8 ± 14.4 μatm higher than those derived from pH-DIC pair. In other words, overestimation of *p*CO_2_ from non-carbonate alkalinity could be up to 6.5 ± 1.4% in Great Lake waters even under the generally low DOC concentrations (Fig. 2). A one-way ANOVA analysis was performed to determine the significance of differences in *p*CO_2_ between different sample groups.

## Additional Information

**How to cite this article**: Lin, P. and Guo, L. Do invasive quagga mussels alter CO_2_ dynamics in the Laurentian Great Lakes? *Sci. Rep.* **6**, 39078; doi: 10.1038/srep39078 (2016).

**Publisher's note:** Springer Nature remains neutral with regard to jurisdictional claims in published maps and institutional affiliations.

## Supplementary Material

Supplementary Information

## Figures and Tables

**Table 1:** Surface water pH, total alkalinity (TA) and dissolved inorganic carbon (DIC), as well as partial pressure of CO_2_ (*p*CO_2_) and air-lake CO_2_ fluxes (F_CO2_) from the Laurentian Great Lakes based on TA-pH and DIC-pH data, respectively.

Station ID	pH	Ca (mg/L)	Mg (mg/L)	TA (μmol/kg)	DIC (μmol/kg)	DIC-*p*CO_2_ (μatm)	DIC-F_CO2_ (mmol-C/m^2^/d)	TA-*p*CO_2_ (μatm)	TA-F_CO2_ (mmol-C/m^2^/d)
**Lake Superior**									
LS-1	8.00	13.9	2.15	850	831	474	2.20	496	2.83
LS-2	7.94	13.8	2.54	850	785	519	3.48	576	5.10
LS-3	8.05	13.9	2.34	840	780	350	−1.32	386	−0.30
LS-4	7.95	13.8	2.25	830	775	502	3.00	551	4.39
AVG	7.98 ± 0.05	13.8 ± 0.1	2.32 ± 0.17	843 ± 10	793 ± 26	461 ± 77	1.84 ± 2.17	502 ± 84	3.01 ± 2.40
1983–2006^30^						500 ± 100			
1996–2006^17^						370 ± 66			
**Lake Michigan**									
LM-1	8.16	32.5	4.61	2180	2107	906	14.48	945	15.59
LM-2	8.21	33.4	3.20	2150	2054	780	10.90	822	12.09
LM-3	8.26	33.4	3.48	2200	2066	691	8.37	739	9.73
LM-4	8.22	32.3	3.02	2160	2053	739	9.73	783	10.98
LM-5	8.25	31.5	2.57	2150	2046	693	8.43	733	9.56
AVG	8.22 ± 0.04	32.6 ± 0.8	3.38 ± 0.77	2168 ± 22	2065 ± 24	762 ± 88	10.38 ± 2.52	805 ± 86	11.59 ± 2.46
1983–2006^30^						480 ± 66			
**Lake Huron**									
LH-1	8.15	26.7	3.04	1680	1605	667	7.69	706	8.79
LH-2	8.07	26.1	2.92	1540	1464	733	9.56	782	10.95
LH-3	8.00	25.5	2.80	1580	1467	875	13.60	961	16.04
LH-4	8.06	32.8	2.96	1600	1540	819	12.01	864	13.28
AVG	8.07 ± 0.06	27.8 ± 3.4	2.93 ± 0.10	1600 ± 59	1519 ± 67	774 ± 92	10.71 ± 2.61	828 ± 109	12.27 ± 3.11
1983–2006^30^						450 ± 330			
1999^39^						317 ± 55			
**Lake Erie**									
LE-1	8.17	31.6	2.76	1860	1771	753	10.13	797	11.38
LE-2	8.16	—	—	1850	1767	768	10.56	810	11.75
LE-3	8.31	25.8	2.42	1880	1784	544	4.19	574	5.04
LE-4	8.12	32.0	2.73	1900	1797	837	12.52	895	14.16
AVG	8.19 ± 0.08	29.8 ± 3.5	2.64 ± 0.19	1872 ± 22	1780 ± 14	725 ± 126	9.35 ± 3.59	769 ± 137	10.58 ± 3.89
1983–2006^30^						400 ± 380			
1999^39^						400 ± 194			
**Lake Ontario**									
LO-1	8.20	32.0	2.64	1790	1694	640	6.92	682	8.11
LO-2	8.24	32.7	2.60	1830	1721	588	5.44	629	6.61
LO-3	8.16	31.9	2.64	1810	1695	700	8.62	755	10.19
LO-4	8.19	32.1	2.64	1830	1710	662	7.54	715	9.05
AVG	8.20 ± 0.04	32.2 ± 0.4	2.63 ± 0.02	1815 ± 19	1705 ± 13	647 ± 47	7.13 ± 1.33	695 ± 53	8.49 ± 1.51
1983–2006^30^						420 ± 200			
**All Great Lakes during 2013**									
	8.14 ± 0.11	27.4 ± 7.4	2.82 ± 0.53	1683 ± 461	1596 ± 442	678 ± 142	8.00 ± 4.02	724 ± 148	9.30 ± 4.20

LS, LM, LH, LE and LO denote Lakes Superior, Michigan, Huron, Erie and Ontario, respectively. AVG denotes averaged values with standard deviation for each lake. Data of *p*CO_2_ between 1983 and 2006 are taken from refs 17, 30 and 39.
