# Supplementary material for: Do invasive quagga mussels alter CO2 dynamics in the Laurentian Great Lakes?
Source: Sci Rep. 2016 Dec 20;6:39078. doi: 10.1038/srep39078 (PMC5171849; doi:10.1038/srep39078)
Supplement: Supplementary Information [file srep39078-s1.pdf]

Do invasive quagga mussels alter CO_2 dynamics in the Laurentian Great Lakes?

Peng Lin [1)] & Laodong Guo*

School of Freshwater Sciences, University of Wisconsin-Milwaukee, 600 East Greenfield Avenue, Milwaukee, WI 53204, USA

* Corresponding author. E-mail: guol@uwm.edu

1) Current address: Department of Marine Sciences, Texas A&M University at Galveston, Galveston, TX 77553, USA.

Key words: Carbon dynamics; *Dreissena,* CO_2 fluxes; Invasive species; Laurentian Great Lakes

Table 1S Comparisons in total alkalinity (TA) and dissolved inorganic carbon (DIC) among Laurentian Great Lakes, open oceans and other lakes.

Location	TA (μmol/kg)	DIC (μmol/kg)
**Laurentian Great Lakes**		
Lake Superior	843±10	793±26
Lake Michigan	2168±22	2065±24
Lake Huron	1600±59	1519±67
Lake Erie	1872±22	1780±14
Lake Ontario	1815±19	1705±13
**Global open oceans**		
North Pacific	2250-2400	2050-2150
Central Pacific	2200-2300	1900-2000
Southern Ocean	2300-2400	2200-2250
North Atlantic	2300-2500	2050-2150
**Other lakes**		
Lake Maggiore[32]	810	900
Lake Washington[33]	643-739	683-793
Loch Ness[34]	45.7	64
